# Venous Thromboembolism and Its Association with COVID-19: Still an Open Debate

**DOI:** 10.3390/medicina56100506

**Published:** 2020-09-27

**Authors:** Pierpaolo Di Micco, Vincenzo Russo, Corrado Lodigiani

**Affiliations:** 1 UOC Medicina, Fatebenefratelli Hospital of Naples, 34102 Naples, Italy; 2 Chair of Cardiology, Department of Translational Medical Sciences, University of Campania “Luigi Vanvitelli”—Monaldi Hospital, Piazzale Ettore Ruggeri, 80131 Naples, Italy; v.p.russo@libero.it; 3 Thrombosis and Hemorrhagic Center, Humanitas Research Hospital IRCC and Humanitas University, 20089 Rozzano, Italy; corrado.lodigiani@humanitas.it

**Keywords:** COVID-19, coronavirus, SARS-CoV2, venous thromboembolism

## Abstract

Asreported by the World Health Organization, a novel coronavirus (COVID-19) was identified as the causative virus of new viral pneumonia of unknown etiology by Chinese authorities on 7 January 2020. The virus was named COVID-19 and because of its ability to cause severe acute respiratory syndrome (i.e., SARS) this infection has also been defined as SARS-CoV2.Furthermore, an association between COVID-19 infection and venous thromboembolism has been reported in several series around the world.For this reason, methods used to improve diagnostic tools, pharmacological thromboprophylaxis and type of anticoagulants are discussed in this expert opinion.

Human coronaviruses (HCoVs) are enveloped non-segmented positive-strand RNA viruses, with rapid evolution owing to their high genomic nucleotide substitution rates and recombination [1]. HCoVs are associated with multiple respiratory diseases of varying severity, including common cold, pneumonia and bronchiolitis. Severe Acute Respiratory Syndrome (SARS) in 2003 [2] and Middle East Respiratory Syndrome (MERS) in 2012 were respiratory infections with high mortality due to HCoVs (2). A highly pathogenic HCoV, named severe acute respiratory syndrome coronavirus 2 (SARS-CoV-2), has been recognized in Wuhan, China, as the cause of the coronavirus disease 2019 (COVID-19) outbreak, with alarming morbidity and mortality [3]. Emerging worldwide clinical experiences testified that COVID-19 showed more lethal action than SARS and MERS [4], probably due to the concomitant alteration of haemostasis with a trend toward microthrombosis and venous thromboembolism (VTE).

Since early epidemiological reports, a hypercoagulable state characterized by increased levels of fibrinogen and D-dimer has been found in hospitalized COVID-19 patients [5,6,7,8]; moreover, an increased rate of pulmonary thrombi and emboli has been found in autoptical series [9], as also shown in other viral pandemicssuch as influenza A H1N1 [10]. Based on these data, clinical researchers focused their attention on life-threatening complications of the hypercoagulable state as pulmonary embolism (PE) or disseminated intravascular coagulation (DIC) [11]. In this way, different clotting abnormalities have been underlined in patients hospitalized for COVID-19 in the ICU compared to other wards, underling that DIC is a serious complication for these patients that can be found early with frequent clotting tests [12]. For this reason, in the literature, some authors reported a positive experience with therapeutic doses of low molecular weight heparin in patients with severe COVID-19 reporting a reduction of mortality and mortality associated withDIC or PE [13].

The prevalence of PE in COVID-19 patients ranges from 15% to 40%; this large difference across clinical studies may be due tothe size and heterogeneity of sample populations, presenting significant differences in clinical characteristics, pre-admission pharmacological treatments and anticoagulation regimens used for VTE prophylaxis during the hospitalization [14,15,16,17]. Moreover, the timing and the methodology to perform VTE diagnosis are heterogeneous across different studies [18,19,20]. Similar counteracting data may be found for the clinical indication to perform thromboprophylaxis with low molecular weight heparin after discharge.

For this reason, the guidelines of the American College of Chest Physicians, in absence of a contraindication, recommend an anticoagulant thromboprophylaxis with low-molecular-weight heparin (LMWH) or fondaparinuxin for hospitalized COVID-19 patients. The routine ultrasound screening for the detectionof asymptomatic deep vein thrombosis (DVT) has not suggested, however, clinicians should have a low threshold for performing ultrasound in patients with a reasonable degree of clinical suspicion for VTE [21]. The use of biomarkers in the diagnostic evaluation for suspected DVT or PE is not suggested. Therefore, an important keypoint is that routinely vascular diagnostics are not suggested because routine prophylaxis is useful and suggested per se during hospitalization for COVID-19. Furthermore, this daily clinical approach is also useful because the routine screening with vascular diagnostics may have misunderstandings in theirapplication: it is still unclear the better time indicated to perform a vascular diagnostic useful to detect asymptomatic VTE in patients affected by COVID-19 (e.g., day 1, 3, 7 or 15 of hospitalization). Similar limitations may be found for symptomatic patients with overt PE: typical signs and symptoms of PE such as dyspnoea and chest pain are also present during COVID-19 per se due to viral and immunological lung injuries, and this similar clinical aspect is associated withthe reduced clinical support given by biomarkers of suspected VTE as d-dimer and alkalosis on haemogasanalysis.

In conclusion, the role of vascular diagnostics for VTE diagnosis in symptomatic or asymptomatic inpatients affected by COVID-19 is still a matter of discussion and needs to be better evaluated as well as the better time to perform vascular diagnostics in order to confirm VTE with objective methods; on the other hand, the useful role of thromboprophylaxis is clear as well as the use of other anticoagulants before hospital admission, because they reduce the rate of VTE in this clinical setting.
